# Radial incision and cutting for post-esophageal endoscopic submucosal dissection stricture with prior perforation during dilation

**DOI:** 10.1055/a-2208-5744

**Published:** 2023-12-11

**Authors:** Jun Takada, Yukari Uno, Kentaro Kojima, Sachiyo Onishi, Masaya Kubota, Takashi Ibuka, Masahito Shimizu

**Affiliations:** 1 Department of Gastroenterology, Gifu University Graduate School of Medicine, Gifu, Japan

Extensive mucosal resection by esophageal endoscopic submucosal dissection (ESD) can lead to strictures 1 . Oral or locoregional steroid injections are used to prevent stenosis, but they are not effective in all cases 2 3 . Endoscopic balloon dilation is the primary option for treating post-ESD strictures 4 ; however, its applicability in previously perforated areas is challenging because of the risk of reperforation. Herein, we present a valuable case of radial incision and cutting for managing a post-ESD esophageal stricture with a prior perforation during endoscopic balloon dilation.

A 76-year-old man with widespread superficial esophageal squamous cell carcinoma underwent ESD, with resection extending over 80% of the esophagus. Despite locoregional steroid injection, a stricture developed. A perforation occurred during endoscopic balloon dilation for stricture management ( Fig. 1 ). Following clip closure and fastening, the perforation healed, but restenosis ensued. Considering the risk of re-perforation, endoscopic balloon dilation was deemed unsuitable; therefore, we decided to perform radial incision and cutting to avoid surgical intervention.

**Fig. 1:**
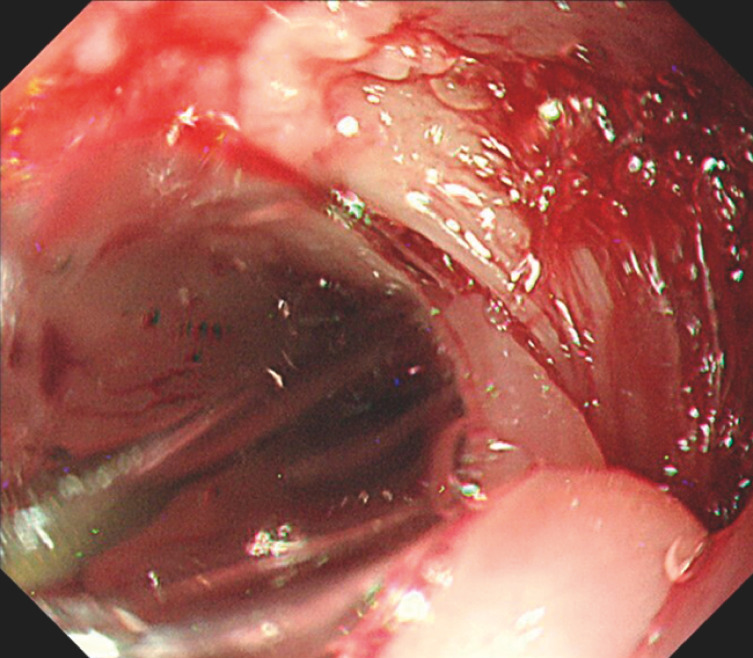
Endoscopic image of the perforation during endoscopic balloon dilation.

The previous perforation site was at the 4 oʼclock position near the mouth side edge ( Fig. 2 ); therefore, the incision was made from different directions using an electrosurgical knife (Dual-Knife J, KD-655Q; Olympus Optical Co., Tokyo, Japan). A cut was carefully made to avoid exposing the muscle layer. A fibrous tissue was removed and the lumen was enlarged until the anal edge of the stricture could be observed, allowing visualization of the direction for safe incision. The incision was advanced in a safe direction viewed from the anal side edge of the stricture to further enlarge the lumen. Finally, the stricture was sufficiently dilated to facilitate easy passage of the scope ( Fig. 3 , Video 1 ). Additional oral and locoregional steroid injections were administered. After 2 months, a scarred radial incision and cutting region without restenosis was observed ( Fig. 4 ).

**Fig. 2:**
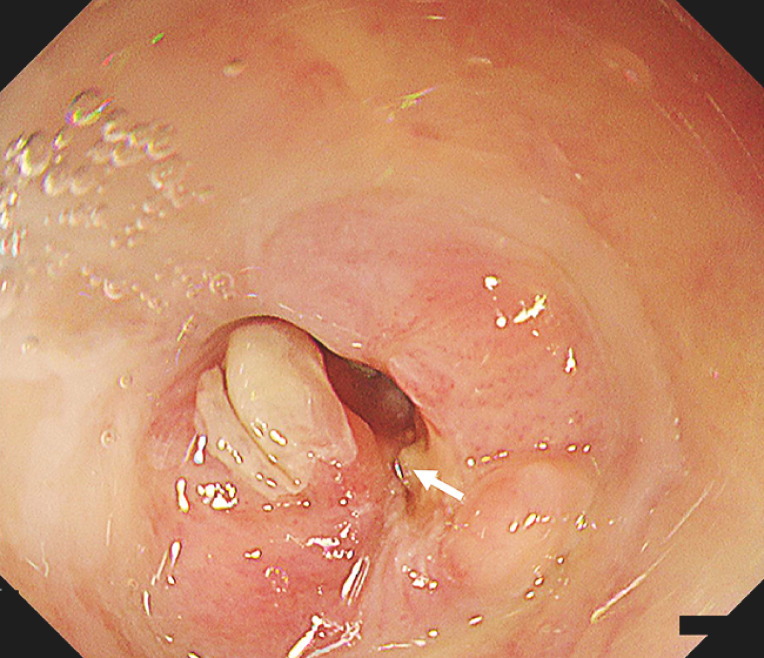
Endoscopic image of the post-endoscopic submucosal dissection stricture before radial incision and cutting. The previous perforation site was identified at the 4 oʼclock position near the edge of the mouth (arrow).

**Fig. 3:**
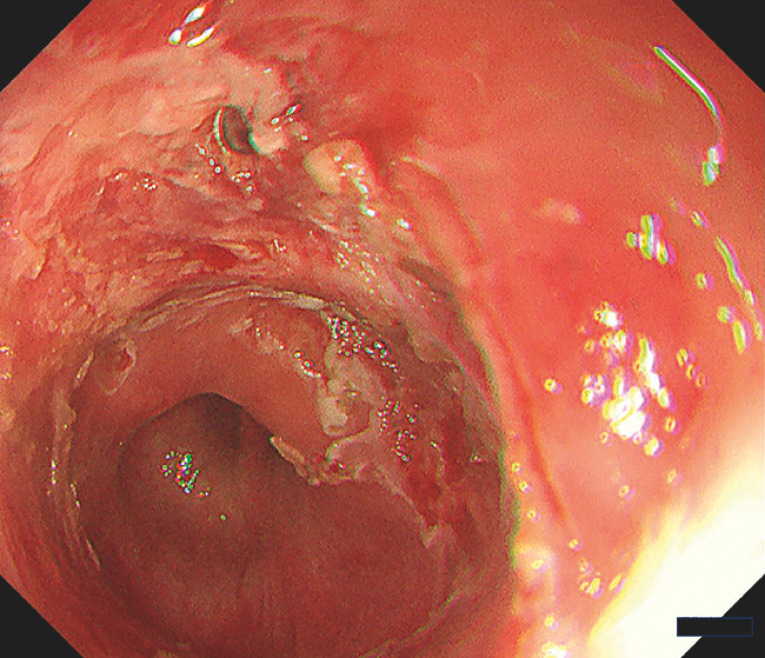
Endoscopic image after radial incision and cutting reveals the head of a buried closing clip at the site of the prior perforation.

Radial incision and cutting of refractory strictures with a prior perforation during balloon dilation after esophageal endoscopic submucosal dissection.Video 1

**Fig. 4:**
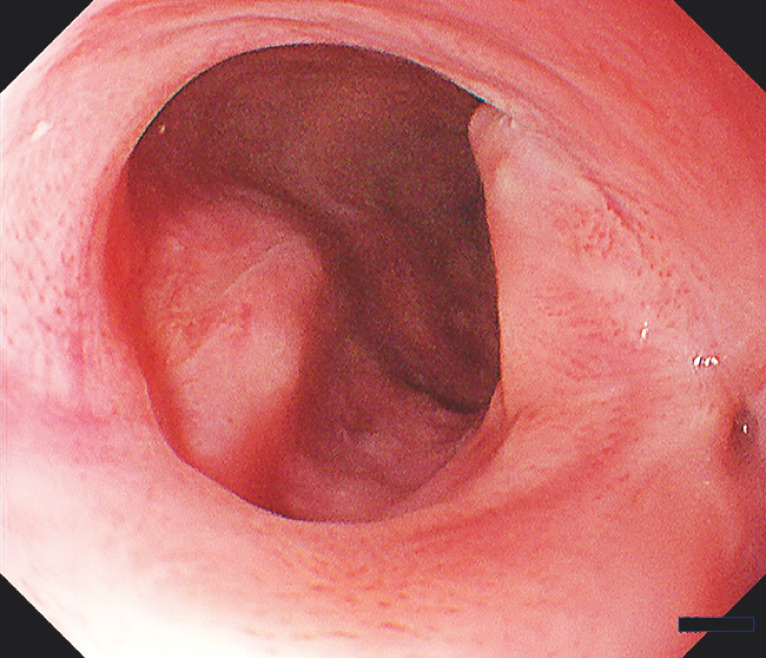
Endoscopic image after 2 months of incision and cutting, displaying a scarred treated region with no signs of restenosis.

Endoscopy_UCTN_Code_TTT_1AO_2AH

## Notes

Endoscopy E-Videos https://eref.thieme.de/e-videos
*E-Videos* is an open access online section of the journal *Endoscopy* , reporting on interesting cases and new techniques in gastroenterological endoscopy. All papers include a high-quality video and are published with a Creative Commons CC-BY license. Endoscopy E-Videos qualify for HINARI discounts and waivers and eligibility is automatically checked during the submission process. We grant 100% waivers to articles whose corresponding authors are based in Group A countries and 50% waivers to those who are based in Group B countries as classified by Research4Life (see: https://www.research4life.org/access/eligibility/ ). This section has its own submission website at https://mc.manuscriptcentral.com/e-videos .
